# Sourcing the origins of carnelian in early Chinese civilizations

**DOI:** 10.1073/pnas.2524563123

**Published:** 2026-02-02

**Authors:** Meiting Yan, Jiancheng Liu, Chunlei Qin, Xuemei He, Xiaoguang Li, Jian Yu, Ruilin Mao, Hongye Han, Zhanwei Sun, Chong Wang, Zhenbin Xie, Honglin Ran, Fei Tang, Jonathan Mark Kenoyer, Zihua Tang

**Affiliations:** ^a^State Key Laboratory of Lithospheric and Environmental Coevolution, Institute of Geology and Geophysics, Chinese Academy of Sciences, Beijing 100029, China; ^b^College of Earth and Planetary Sciences, University of Chinese Academy of Sciences, Beijing 100049, China; ^c^Sichuan Provincial Institute of Cultural Relics and Archaeology, Chengdu 610041, China; ^d^Beijing Bead Museum, Beijing 100024, China; ^e^School of Gemmology, China University of Geosciences, Beijing 100083, China; ^f^State Key Laboratory of Lithospheric Evolution, Institute of Geology and Geophysics, Chinese Academy of Sciences, Beijing 100029, China; ^g^Sanxingdui Museum, Guanghan 618300, China; ^h^Gansu Provincial Institute of Cultural Relics and Archaeology, Lanzhou 730015, China; ^i^Beijing Institute of Archaeology, Beijing 100009, China; ^j^Shaanxi Academy of Archaeology, Xi’an 710075, China; ^k^University of Wisconsin-Madison, Madison, WI 53706

**Keywords:** carnelian beads, sourcing, trace elements, Chinese Bronze Age, Sanxingdui

## Abstract

Carnelian beads in high-status burials of the Western Zhou period (ca. 1000–800 BCE) have long been seen as key evidence for long-distance exchange between East Asia and regions to the west, while their geological origins and circulation pathways have remained poorly constrained. Using a newly established geochemical database of 300 geological samples from 27 potential sources across Asia, we conducted trace-element analyses of 11 carnelian beads from the Sanxingdui pits (ca. 1200–1000 BCE), Sichuan Basin, southwest China. Canonical discriminant analysis indicates that the raw materials of these carnelian beads do not primarily derive from south China, but the Yanshan Orogeny, Central Asian Orogenic Belt and some unknown sources that might be close to Hexi Corridor, pointing to raw-material sources located over 1,000 km to the north of the Sichuan Basin. Comparative analyses of contemporaneous beads from Gansu, Shaanxi, and Beijing show similar northern provenance signatures, suggesting a broad and persistent exchange sphere spanning the southern Mongolian Plateau, Loess Plateau, eastern Tibetan Plateau, Central Plains, and Sichuan Basin between 1500–1000 BCE. Our results provide the earliest direct geochemical evidence for long-distance carnelian exchange in Bronze Age China and demonstrate the value of integrating geochemical sourcing with archaeological context to reconstruct ancient interaction networks.

## Significance

Understanding the origin of materials in prehistoric ornaments is vital for studying ancient trade and interaction networks. This research presents the large-scale, consistently analyzed geochemical reference database for carnelian in East Asia, encompassing 27 geological sources from diverse regions. Using laser ablation inductively coupled plasma mass spectrometry (LA-ICP-MS) trace element fingerprinting and canonical discriminant analysis, we achieved over 90% accuracy in identifying carnelian origins. Applying this method to beads from Sanxingdui and other contemporary sites reveals extensive long-distance material networks linking the Sichuan Basin with northern China. These results highlight significant interregional exchanges and offer a universal protocol for sourcing microcrystalline quartz artifacts worldwide.

Many early scholars have argued that carnelian was not a traditional material for personal adornment in China (1, 2) during the Neolithic or early Bronze Age. Its sudden proliferation in high-status burials in the late Bronze Age during the early Western Zhou period has been linked to the expansion of exchanges between the Central Plains and the Eurasian interior and to the introduction of new materials and aesthetic preferences (2, 3). Although earlier studies of carnelian from Western Zhou burials were thought to have come from West Asia (4) or were local production imitating foreign styles (5), this interpretation is no longer tenable due to technological studies of the beads themselves and new discoveries of local bead production in Gansu (6, 7) and southwest Inner-Mongolia (8). While some scholars still assume that carnelian was a novel decorative material to China (9), as will be demonstrated in this paper, its local sourcing and use during earlier periods is evidence that this material has long been used as a marker of social hierarchy and cultural identity.

Earlier scholarship generally characterized Bronze Age China as centered on the Yellow River valley, distinguished by the close association of bronze artifacts with kingship, social ranking, and order (10, 11). With the exploitation of metal ores, peripheral regions—including the middle and lower reaches of the Yangtze River—were gradually integrated into the cultural sphere of the Central Plains (10). The Sanxingdui site, located in the Sichuan Basin, has reshaped this narrative. Sanxingdui is a major Bronze Age center in southwest China (ca. 1700 to 1000 BCE), contemporaneous with but culturally distinct from the Erligang and Late Shang traditions of East China. Its ritual system is characterized by large bronze masks, gold objects, and finely worked jades, reflecting the emergence of an independent regional polity within wider East Asian interaction networks (12, 13). The eight special object burial pits with lots of ritual related items—extraordinary deposits of intentionally broken or burnt high-value items such as bronzes, ivory, gold, cowries, and carnelian—represent concentrated episodes of elite ritual activity around 1200 to 1000 BCE and provide a rare, well-defined context for studying materials circulating within the highest levels of Sanxingdui society (14). Carnelian beads occur almost exclusively in these pits, underscoring their special significance and making them ideal indicators of long-distance exchange. By integrating provenance data with this archaeological context, we directly illuminate the scale, direction, and nature of Sanxingdui’s external connections at a pivotal stage in early state formation in the upper Yangtze region. In the absence of any written records, tracing the sources and roles of these materials is essential to understanding the foundations of this prosperous and technologically sophisticated society; without such knowledge, the origins of political authority in this still poorly understood region remain obscure. The 11 carnelian beads, dating to ca. 1200 to 1000 BCE (12), are the southernmost known examples of carnelian artifacts from this period in China and provide crucial evidence for reconstructing the origins and dispersal of carnelian materials and beads in East Asia. This finding sheds light on Bronze Age trade networks and cultural interactions in China.

Most recently, the combined use of LA–ICP–MS analysis of carnelian composition and statistical provenance methods has been successfully applied in South Asia (15), the Mongolian Plateau (16), and Southeast Asia (17). In this study, we analyzed the elemental compositions of the carnelian beads from the Sanxingdui pits and, using the same methodology as in the regions mentioned above, established a comprehensive geochemical database of carnelian sources in eastern Asia and South Asia. We defined compositional criteria for distinguishing major geological source areas and applied statistical analyses to determine the provenance of the Sanxingdui specimens. Building on these results, we integrate contemporaneous provenance data for carnelian beads from four sites in China to explore patterns of cultural interaction during the Bronze Age.

## Results

### Mineralogical and Compositional Analyses of Carnelian Beads from the Sanxingdui Pits.

All carnelian beads from the Sanxingdui pits are a form of chalcedony and exhibit the characteristic Raman peak of α-quartz at 464 cm^−1^, which corresponds to the strongest Raman mode assigned to the vibration of SiO_4_ six-membered rings (18). A shoulder at 502 cm^−1^ corresponds to the symmetric stretching vibration of four-membered Si–O–Si rings, previously demonstrated in Raman studies to indicate the presence of moganite (18–20) (see Dataset S1 for further details).

Elemental compositions of the beads were determined by LA–ICP–MS, with ten selected analytical spots per specimen, characterizing a total of 57 elements (see *Materials and Methods*; data provided in Dataset S2). LA–ICP–MS results show that the archaeological samples from Sanxingdui consist of more than 98 wt% SiO_2_ with compositional features consistent with carnelian.

**Fig. 1.**
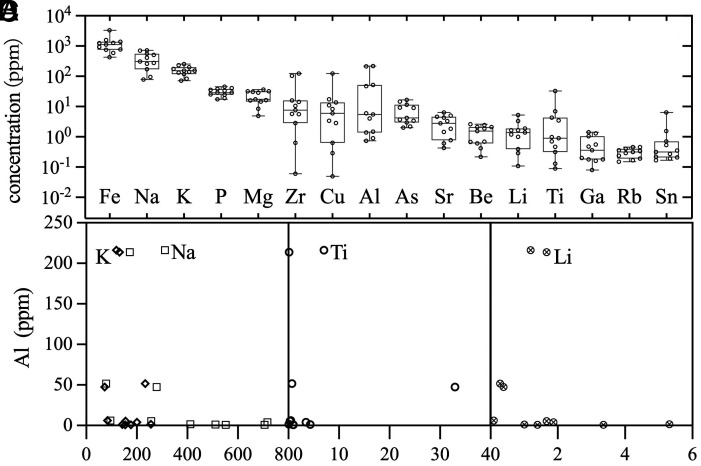
Trace element compositions of carnelian beads from Sanxingdui. (*A*) Chemical variability of the carnelian beads. K, Na (*B*), Ti (*C*), and Li (*D*) versus Al concentrations from the carnelian beads of Sanxingdui.

Trace-element concentrations vary markedly among the Sanxingdui specimens (Fig. 1). Fe contents are approximately 1,000 ppm, while several beads (e.g., specimens Sc01 and Sc02) exhibit elevated Al contents (>200 ppm). In addition, Ti concentrations in most Sanxingdui carnelian beads are below 20 ppm, consistent with a hydrothermal origin (21).

### Geological Source Database and Geochemical Characterization of Carnelian.

Establishing a robust database of carnelian sources and maximizing differentiation among source regions are prerequisites for provenance analysis. We collected carnelian samples from 27 potential source regions, covering major tectonic units including the Central Asian Orogenic Belt (n = 5), the North China Craton (n = 6), the Qinling-Dabie orogenic belt (n = 2), the South China Block (n = 7), and the South Asian subcontinent (n = 7). From each potential source region, 2 to 22 specimens were collected and analyzed for elemental composition using LA-ICP-MS. For each specimen, two selected spots were measured, yielding concentrations for 57 elements under the same analytical conditions used for the archaeological samples (see Dataset S3 for details). This is currently the most extensive compositional database of carnelian raw materials in China and its surrounding regions, and it is still being expanded. We will continue to update this database with additional specimens from a wider range of locations.

Based on compositional differences among source-region samples (Dataset S3), canonical discriminant analysis (CDA, see *SI Appendix* for further details) grouped the 27 potential sources into four compositional provinces corresponding to similar geological backgrounds and geographic proximity: South Asia (SA), South China (SC), the Central Asian Orogenic Belt (CAOB), and the Yanshan Orogeny (YSO). The canonical discriminant model used 20 elements—Li, Be, Na, Mg, Ca, V, Mn, Fe, Cu, Ga, Sr, Y, Zr, Sn, Sb, Cs, Ba, Pr, Pb, and U—achieving a classification success rate of 92.3% for the original grouped cases and 90.3% for cross-validation. This high level of classification accuracy makes it feasible to determine the provenance of raw material of the Sanxingdui carnelian beads based on their elemental compositions.

ANOVA results indicate significant compositional differences among the four source regions (see *SI Appendix* for details). In brief, SA carnelian samples are characterized by high Li and Ga and low Be, Cs, and U contents; SC samples show relatively high Cu and Sr but low Li and Pb; CAOB samples contain relatively high Be, Sb, and Ba; and YSO samples have notably high U contents. These elemental patterns constitute distinct geochemical fingerprints for each source region.

### Provenance Results for Archaeological Samples.

**Fig. 2.**
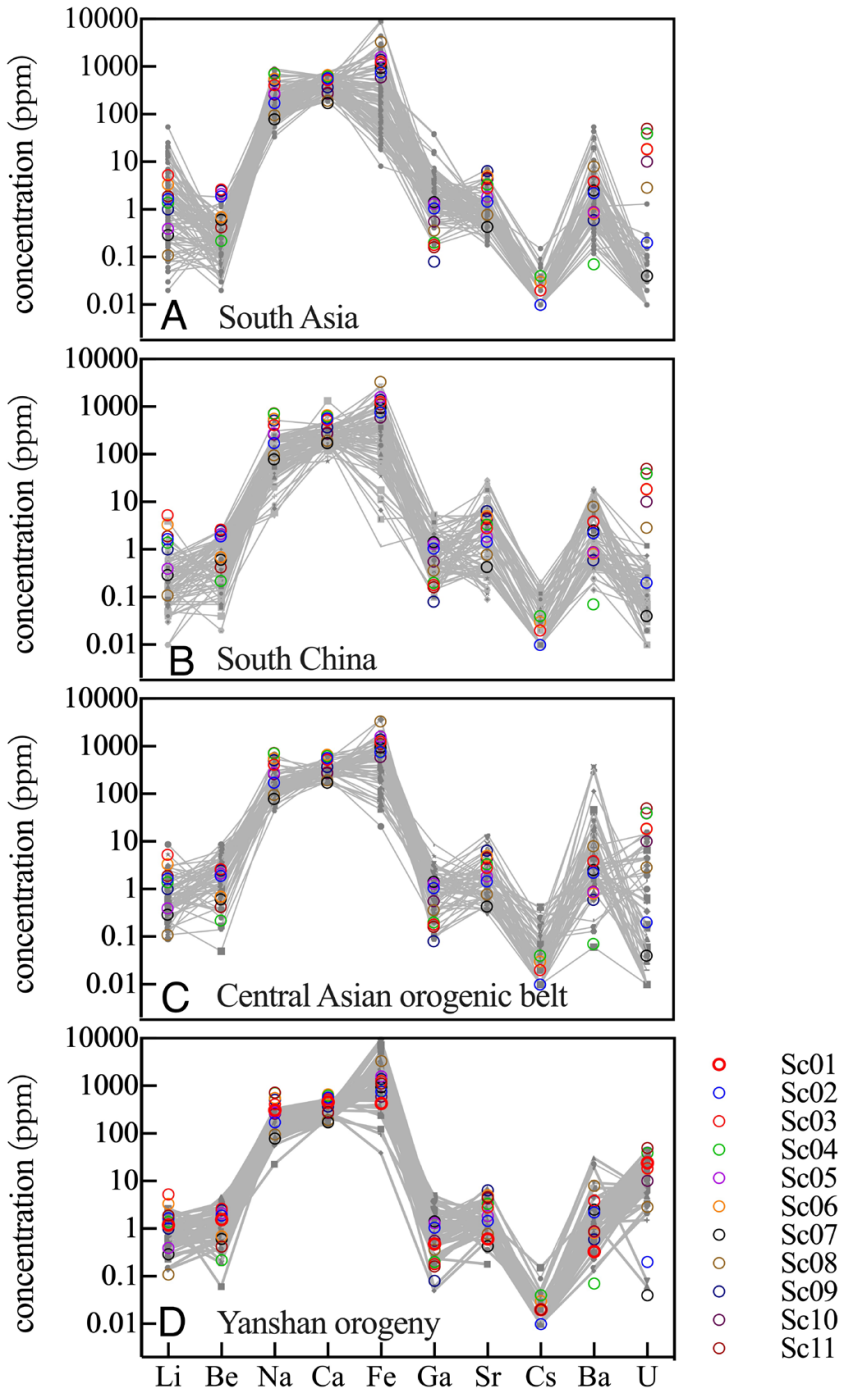
A comparison of Sanxingdui samples and geological specimens from SA (*A*), SC (*B*), CAOB (*C*), YSO (*D*) based on trace-element profiles. The gray lines represent the observed values of geological specimens from various source provinces, while the colored dots represent the compositions of the carnelian beads from Sanxingdui.

The trace-element composition of chalcedony is not constrained by the quartz lattice, and its geochemical variability can be used to identify diagnostic features of individual samples. Using elements that exhibit pronounced differences among source regions, we compared the trace-element profiles of the Sanxingdui specimens with the characteristic geochemical signatures of the four defined source provinces (Fig. 2). In geochemical provenance studies, “matching” chemical fingerprints typically involves comparing the shape and relative abundances of trace-element patterns in archaeological specimens with those of potential source rocks to establish a likely origin.

As shown in Fig. 2, the Sanxingdui carnelian differs markedly from South Asian samples in U contents, indicating that South Asia is an unlikely primary source. On similar grounds, the South China source can also be excluded as the main provenance. Notably, while diagnostic trace elements can eliminate certain source regions, they are not sufficient on their own to identify a single definitive origin.

### Provenance Classification Based on Canonical Discriminant Analysis.

We applied canonical discriminant analysis (CDA) to the Sanxingdui carnelian data together with the potential source dataset, using the first predicted group (1st PGM) and second predicted group (2nd PGM) assignments to assess provenance. The 1st PGM represents the group most similar to the unknown sample, while the 2nd PGM indicates the second closest match. One important aspect to note is that CDA assumes that a dataset being evaluated contains all possible groups. As a result, CDA does assign a predicted group membership (PGM) to each unknown specimen in the existing database. Of the 11 carnelian beads from the Sanxingdui pits, seven map closest to the YSO field in the discriminant space and are therefore provisionally assigned to the Yanshan province. Three specimens (Sc02, Sc07, Sc10) plot nearer to the Central Asian Orogenic Belt field and are assigned to the broader CAOB group. One specimen (Sc08) falls between defined clusters and shows mixed posterior probabilities (~75% South Asia, ~25% CAOB). However, its elemental pattern—particularly Cs, Ga, Sr, U, and Pb—clearly excludes the South Asian group and aligns it with the northern orogenic fields as a whole. We therefore treat Sc08 as most consistent with an unsampled or compositionally variable segment of the CAOB.

Notably, several archaeological specimens (mainly from Mogou site) fall at the margins rather than the centers of their assigned geological clusters, suggesting that their classification reflects broad regional affinity rather than a strict match to currently sampled deposits. Expanded geological sampling will likely refine these assignments and may reveal additional northern sources not yet represented in the reference dataset.

## Discussion

### Sourcing Potential of the Carnelian Geological Database.

Trace-element provenance analysis of carnelian has been successfully applied in Southeast Asia (15) and Mongolia (16), but previous studies lacked extensive geological samples from China, leaving existing source databases incomplete. Here, we established a compositional database covering eastern Asia using a standardized analytical protocol and consistent quality control, and we identified the YSO as a distinct source province. This provides a robust foundation for sourcing carnelian using trace-element geochemistry.

Canonical discriminant analysis (CDA) of 300 geological specimens from 27 potential sources, refined through multiple iterations, yielded a well-resolved classification into four provinces: SA, SC, CAOB, and YSO. The classification accuracy was 92.3% for the original grouped cases and 90.3% for cross-validation, comparable to the success rates reported in previous provenance applications (16). These results demonstrate the potential of our database to determine the provenance of unknown carnelian samples.

Notably, the four provinces defined by CDA correspond to markedly different geological backgrounds. From north to south: the CAOB formed through the progressive accretion of terranes between the Siberian Craton and the North China Craton (22); the YSO was generated during Mesozoic subduction of the Paleo-Pacific Plate, where partial melting of the subduction zone and crustal anatexis provided new magmatic sources (23); carnelian in South China, particularly from Liangshan and Baoshan, occurs around the margins of a Late Paleozoic large igneous province (24); and carnelian deposits in India are closely related to the evolution of the Deccan Large Igneous Province (25). Given that carnelian composition primarily reflects regional volcanic and hydrothermal processes, potential source provinces defined within the framework of magmatic evolutionary history may carry clearer geological significance—a point that warrants further investigation.

Although CDA assigns most Sanxingdui and Mogou beads to the YSO and CAOB provinces, their positions in the discriminant space do not fully overlap with the tight geological clusters from these provinces. The archaeological specimens appear to be placed by relative exclusion of the South China and South Asia regions rather than by perfect similarity to any currently sampled deposit. We interpret this as evidence of two limitations in the present geological baseline: i) Carnelian from large orogenic provinces is compositionally heterogeneous, and ii) some potential northern deposits remain unsampled.

Within these constraints, the beads show consistent elemental patterns—especially in Cs, U, Ga, Sr, and Pb—that robustly rule out southern sources and align the artifacts with the broader northern orogenic systems. Their slight offsets from sampled clusters likely reflect uncharacterized deposits within the same geochemical provinces rather than alternative southern origins. This possibility underscores the need for expanded geological sampling while strengthening the regional-scale conclusion: The Sanxingdui and Mogou assemblages are compositionally tied to northern carnelian provinces, supporting sustained north–south interaction networks during the second millennium BCE.

Our study presented here is designed to trace the geological origins of raw material of carnelian beads; the locations where carnelian beads were actually manufactured remain an open question. Limited investigations consistently pointed to the Hexi Corridor and southwest Inner-Mongolia as key production zones, including the western site of Ganguya (6), the eastern site of Huoshitan in Minqin at the corridor (26) and northern sites of Mantissar, Gurnai Depression, and Ukh-tokhoi cave site in Alashan Gobi Desert (8). Future research on raw-material sourcing and bead-making technologies in the Hexi Corridor will hopefully advance our understanding of carnelian provenance and the transmission of related craft traditions.

### Sanxingdui and Its Connections with Northern China.

The provenance results from raw material of Sanxingdui carnelian beads support the idea that, during the Bronze Age, the Sichuan Basin was linked to a broad expanse of northern China, including the Mongolian Plateau and the North China Plain. This inference is independently corroborated by archaeological (13, 27, 28), genetic (29), and linguistic evidence (30, 31). The Sanxingdui site and the two identified source regions are linked through the conspicuous personal ornament of carnelian beads, suggesting that ca. 3000 y ago, Sanxingdui community benefited from a long-distance interaction network extending to northern China and possibly the Mongolian Plateau.

**Fig. 3.**
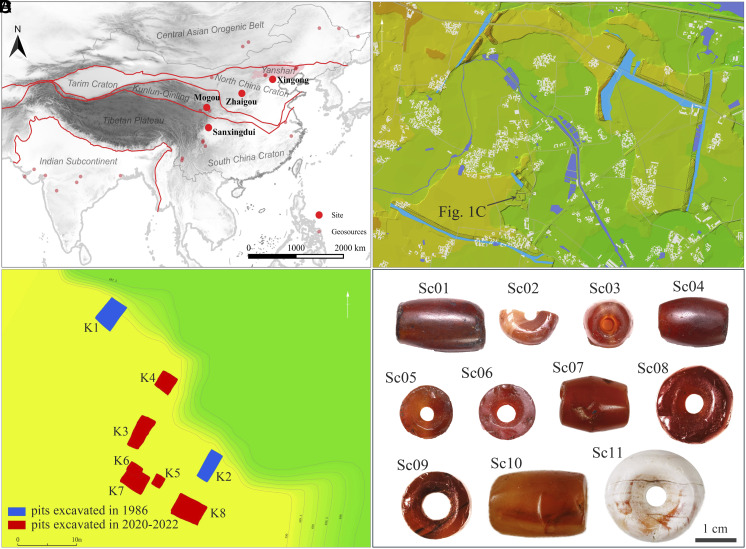
Location of the Sanxingdui site. (*A*) The Sanxingdui site, the related sites and the investigated sources for carnelian materials. Red lines mark the boundaries of tectonic units, which comprise of, from north to south, Central Asian orogenic belts, Tarim-North China Craton, Kunlun-Qinling-Dabie orogeny, Tibetan-Plateau-South China Block, and Indian subcontinent; (*B*) Plan of the Sanxingdui site; (*C*) Planar distribution of the Sanxingdui pits, K1–K8; (*D*) Carnelian beads excavated from the pits.

To better constrain the carnelian exchange networks of the Sanxingdui period, we analyzed extra carnelian beads dating to 1500 to 1000 BCE from the Mogou site in Lintan County, Gansu (32, 33), the Zhaigou site in Qingjian County, Shaanxi (34), and the Xingong site in Beijing (35) (Fig. 3*A*). These beads were mainly sourced from either YSO or the CAOB. During 1500 to 1000 BCE, sites across northern China consistently used carnelian from the same source regions for personal ornaments, indicating both sustained long-distance contacts and the existence of a material culture exchange network.

It is the degree to which social actors monopolize the sources of core power—agricultural production, the flow of prestigious goods, and the use of legitimate violence that determine whether a society is relatively equal or highly hierarchical (36). The intricate layout of the Sanxingdui city walls, the exceptional concentration of valuables in the pits, and the specialized functions of ritual bronzes collectively point to a complex society with a high degree of centralized authority (37, 38). This interpretation is further supported by recent archaeological research, which identifies the emergence of an early state at Sanxingdui based on analyses of settlement morphology, spatial organization, and variations in material assemblages across different communities (39). It is widely recognized that building and maintaining long-distance interaction networks was an important strategy for elites in early complex societies to acquire and consolidate political power (40, 41). At Sanxingdui, delicate carnelian beads only occurred alongside large bronze vessels, gold artifacts, and jade objects in the pits, suggesting that these high-value goods may have jointly served as instruments of elite authority. Such long-distance interaction network not only provided access to high-value goods that were geographically and socially distant from commoners, but the act of exchange also served as a visible performance of status and prestige, reinforcing elite social distinction (42).

The present study represents the attempt at geochemical sourcing of carnelian in China. Although the number of sites analyzed remains limited and is insufficient to reconstruct the precise route by which these carnelian beads entered the Sichuan Basin, the absence of carnelian beads from contemporaneous Shang-period sites in the middle Yangtze River valley allows us to tentatively rule out the route via the middle Yangtze that has previously been proposed for the movement of jade and bronze vessels into Sanxingdui (14, 43, 44).

Moreover, all four sites considered here predate Rawson’s observation for the Western Zhou period (4) and used exclusively northern Chinese carnelian sources, suggesting that the local adoption of carnelian materials began earlier than previously recognized. The timing of carnelian’s introduction and the history of local material adoption remain to be clarified with additional archaeological evidence.

## Conclusion

This study establishes the most comprehensive geochemical database for carnelian in China to date, built under consistent analytical protocols and quality controls. By incorporating previously unavailable Chinese geological samples, we differentiate major tectonic provinces—including the Yanshan Orogeny and the Central Asian Orogenic Belt—and achieve high classification accuracy (>90%) across 300 specimens from 27 potential sources. These results demonstrate the strong sourcing potential of this framework while also revealing geochemical overlaps and boundary cases that reflect the natural heterogeneity of large orogenic systems.

Geochemical analyses of carnelian beads from the Sanxingdui pits indicate predominant affinities with northern Asian sources, especially the Yanshan and Central Asian orogenic belts. Although several specimens occupy marginal positions between clusters, their elemental compositions collectively exclude South China and South Asia as likely sources and instead support a broader northern provenance. These results provide material evidence for sustained connections linking the Sichuan Basin with northern China and the Mongolian Plateau during the late second millennium BCE.

Comparative analysis of contemporaneous beads from Gansu, Shaanxi, and Beijing shows consistent reliance on northern raw materials across widely separated regions, reflecting shared source choices and long-distance exchange networks. The absence of carnelian from Middle Yangtze Shang-period sites, combined with early adoption of northern materials, challenges the hypothesis of a Middle Yangtze entry route into the Sichuan Basin.

Our findings refine current understandings of Sanxingdui’s external interactions and illustrate the interpretive power of integrating geochemical sourcing with archaeological, genetic, and linguistic evidence. Continued expansion of geological sampling—particularly within northern Asia—will further constrain provenance assignments and clarify the trajectories and sociopolitical roles of exotic materials in early complex societies.

## Materials

### Archaeological Specimens.

Carnelian beads from the Sanxingdui pits (n = 11), Mogou site (n = 8), Xingong site (n = 3), and Zhaigou site (n = 2) were analyzed for provenance determination.

### Geological Samples.

Geological specimens were collected from 27 potential source areas in China, India, Mongolia, and Bangladesh. Based on tectonic units, these include the Central Asian Orogenic Belt (five source areas, 45 specimens), North China Craton (six source areas, 60 specimens), Kunlun–Qinling–Dabie Orogenic Belt (two source areas, 29 specimens), Yangtze–Cathaysia Block (seven source areas, 61 specimens), and the South Asian subcontinent (seven source areas, 105 specimens). Detailed information can be found in the *SI Appendix*, *Dataset*.

## Methods

### Raman Spectroscopy.

Raman analyses were conducted at the Institute of Geology and Geophysics, Chinese Academy of Sciences, using a WITec alpha 300R confocal micro-Raman spectrometer (Germany) at room temperature. A 50× Zeiss objective lens (NA = 0.75) was used to focus the laser beam onto the sample surface, and Raman data were calibrated against the silicon peak at 520.7 cm^−1^. Spectra were acquired with a 532 nm excitation laser, an integration time of 2 s, and 40 accumulations per measurement. A 300 g/mm grating was used to cover the spectral range of 100 to 4,500 cm^−1^. A minimum of five measurements were taken for each sample.

### LA-ICP-MS Analysis.

In situ trace element analyses of carnelian beads from the Sanxingdui pits and potential geological source materials were performed using an Analytik Jena PlasmaQuant mass spectrometer coupled to a RESOLution 193 nm excimer laser ablation system. Ablation was conducted with a 72 μm spot size, 5 Hz repetition rate, and ~6 J/cm^2^ energy density, using high-purity helium as the carrier gas. Five reference materials (NIST 610, NIST 612, BHVO-2G, BCR-2G, and BIR-1G) were employed for calibration. Before analyses, the instrument was tuned with NIST 610 to achieve optimal performance. A single-spot ablation mode was applied, with 20 s of background acquisition followed by 45 s of continuous sample ablation and a 20 s washout period. One set of standards was inserted after every 10 ablation spots for quantification. Ten random points were analyzed on each artifact bead, and two points were analyzed on each geological specimen, with 57 elements measured per spot.

### Statistical Analysis.

LA-ICP-MS data reduction was carried out using ICPMSDataCal software. After preprocessing, cleaning, and normalization, statistical analyses were performed with IBM SPSS Statistics 26. Canonical discriminant analysis (CDA) was applied to distinguish between archaeological and geological samples. CDA uses multivariate data to maximize separation among predefined groups (45); in this study, the groups consisted of geological samples from individual agate deposits. Wilks’ Lambda stepwise method was used to select the most discriminative elements for source differentiation. As for the statistical parameters, we chose the F value as the criteria for the stepwise method, with 3.84 as F entry value while 2.71 as F removal value. We assumed all groups were equal for the prior probabilities. Within-groups covariance matrix was used for the statistical procedure.

**Fig. 4.**
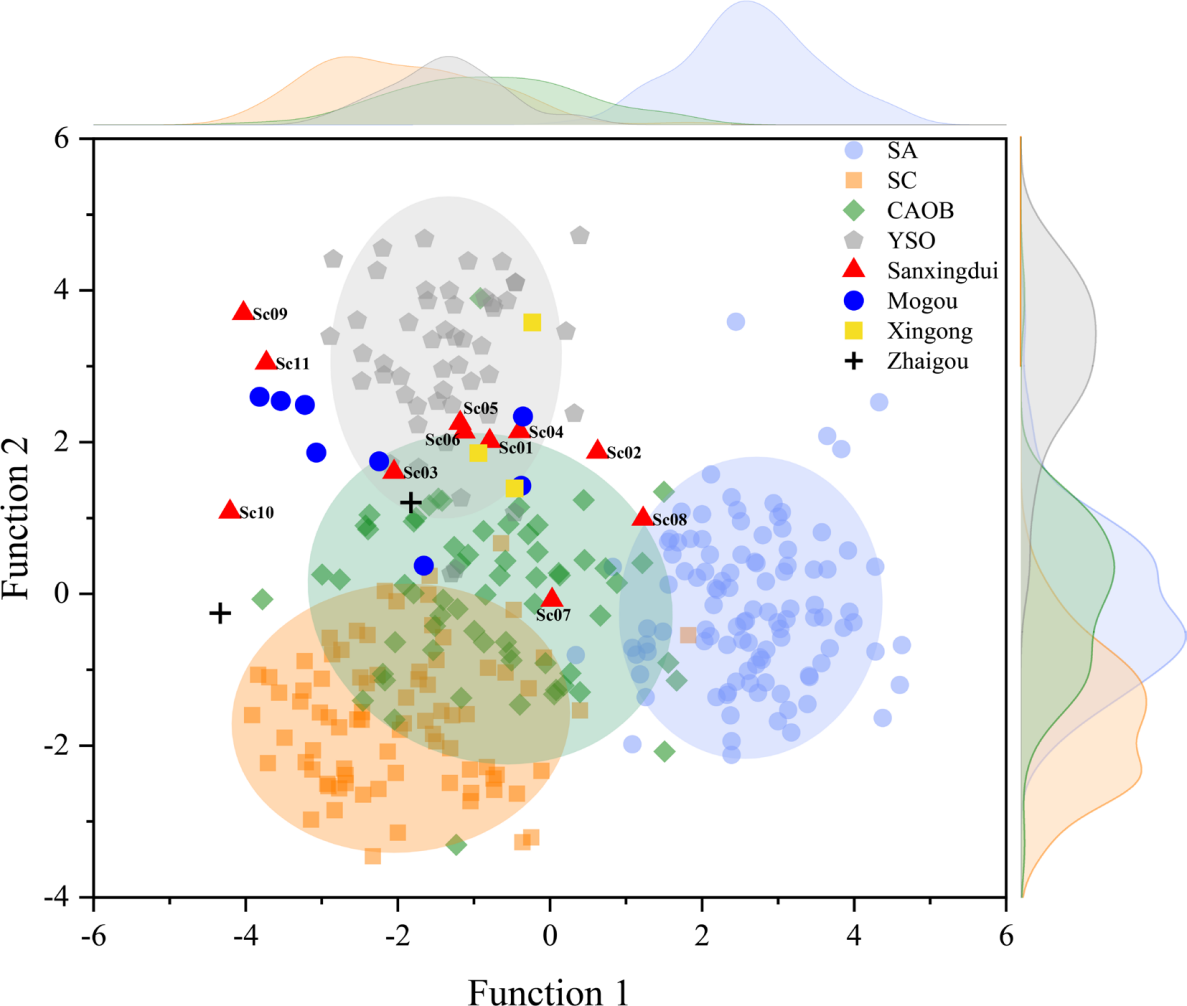
Carnelian beads from Broze Age China compared to major source areas. This plot is based on CDA results, projecting multiple chemical variables into two dimensions. Each colored ellipse represents a range for different given source province: blue for South Asia (SA), orange for South China (SC), green for the Central Asian Orogenic Belt (CAOB), and gray for the Yanshan Orogeny (YSO).

Based on the discriminant functions, artifacts (agate/chalcedony beads) were treated as ungrouped cases and assigned to predicted group memberships (PGMs) according to their Mahalanobis distances to group centroids in multidimensional space. The 1st PGM represents the group most similar to the unknown sample, and the 2nd PGM represents the second closest match (Fig. 4).

## Supplementary Material

Appendix 01 (PDF)

Dataset S01 (XLSX)

Dataset S02 (XLSX)

Dataset S03 (XLSX)

Dataset S04 (XLSX)

## Data Availability

Study data are included in the article and/or supporting information.
