# Supplementary material for: Sourcing the origins of carnelian in early Chinese civilizations
Source: Proc Natl Acad Sci U S A. 2026 Feb 2;123(7):e2524563123. doi: 10.1073/pnas.2524563123 (PMC12913004; doi:10.1073/pnas.2524563123)
Supplement: Supplementary file 1 — Appendix 01 (PDF) [file pnas.2524563123.sapp.pdf]

Supporting Information for Yan et al.

Supplementary Dataset

Dataser S1 Raman spectra data of Sanxingdui carnelian artefacts

Dataset S2 Archaeological sites where carnelian artifacts mentioned in this study were unearthed (Site info); excavation units and age of carnelian artifacts (Unit & Date), elemental composition data (LA-ICPMS), and canonical discriminant analysis results (CDA)

Dataset S3 Abbreviations, full names, number of specimens, locations, and countries of raw material sources (sheet Sources); composition of raw materials (sheet Composition of raw materials)

Dataset S4 Mean of elemental composition for grouped source regions

Supplementary Materials

Regional geological background of potential source areas

Chalcedony, a microcrystalline variety of quartz, occurs in several forms, among which carnelian is a distinctive variant(1). Its formation is closely associated with volcanic activity, magmatic-hydrothermal processes, and related geological events, making it a direct product of such activity. Carnelian is most commonly found in volcanic rocks but can also occur in sedimentary contexts(2). Variations in mineralization conditions can influence the geochemical signatures of carnelian, which are inherently linked to the geological background of their formation.

The tectonic framework of the Asian continent was shaped by interactions among the Eurasian, African, Pacific, and Indo-Australian plates. It consists of six large to medium-sized cratons and five major active tectonic belts located between or along the margins of these cratons(3). China and adjacent regions are mainly composed of the stable Indian craton, the North China craton, and the Yangtze craton, together with intervening active tectonic belts such as the Central Asian Orogenic Belt and the Kunlun–Qinling–Dabie Orogenic Belt (Fig. S1).

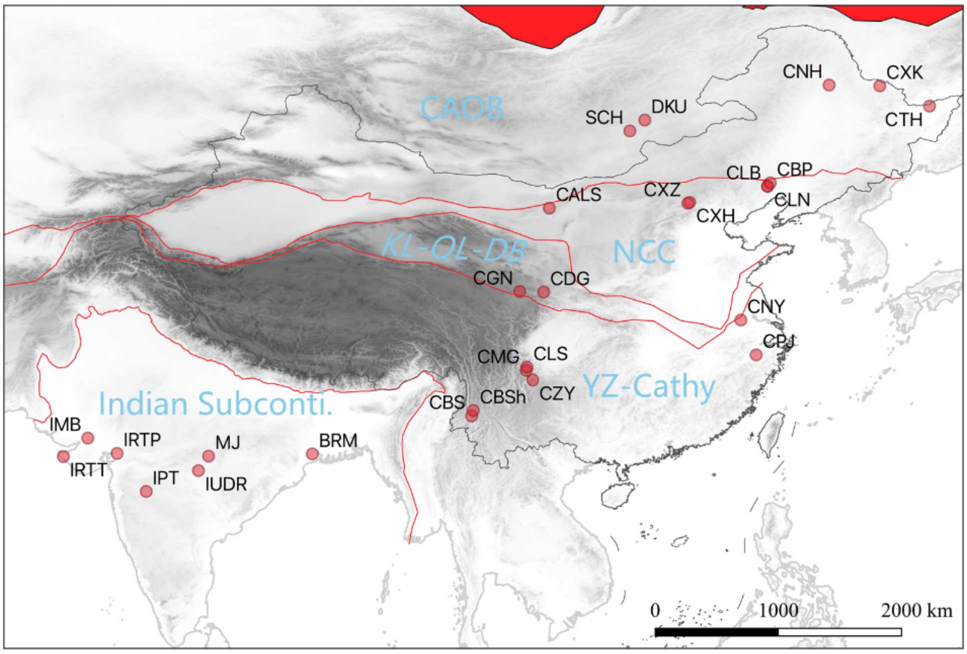

Fig S1. The tectonic units in eastern part of China and the locations of the investigated sources of carnelian raw material. CAOB, Central Asian Orogenic Belt. It is also known as the Altai-Mongolia-Central Asia Orogenic Belt, an accretionary orogen formed during the closure of the Paleo-Asian Ocean. It developed through the progressive accretion and amalgamation of multiple geological entities; NCC, North China Craton. It was the oldest stable continental nucleus in northern China and, in the Mesozoic, underwent significant modification due to the Yanshanian movement induced by the westward subduction of the paleo-Pacific plate; KL-QL-DB, the Kunlun-Qinling-Dabie orogeny. The evolution of this orogenic belt spanned from the Neoproterozoic to the Mesozoic, forming belts of high-pressure and ultra-high-pressure metamorphic rocks; YZ-Cathy, Yangtze and Cathaysia blocks in South China; Indian subconti., Indian subcontinent.

The Indian craton forms the core of the South Asian subcontinent and represents an exotic terrane derived from the breakup of Gondwana(4).
The North China craton is characterized by widespread Yanshanian intrusions and volcanic rocks east of the Taihang Mountains. Intense tectonomagmatic activity in this region created the Yanshan tectonic domain, a distinctive intracontinental zone of strong deformation and magmatism(5).
The Yangtze craton, located in southern China, is a stable block that, during the early Neoproterozoic Rodinia supercontinent assembly, merged with the Cathaysia block to form the unified South China plate(6).
The Central Asian Orogenic Belt (CAOB) is a giant suture zone formed mainly by the subduction and closure of the Paleo-Asian Ocean between the Siberian craton and the North China and Tarim cratons. It is the largest and most structurally complex tectonic domain in the Asian continent(7).
The Kunlun-Qinling-Dabie orogenic belt is an intercontinental orogen situated between the Tarim, North China, and Qiangtang-Yangtze cratons. It represents both a crustal accretional and convergent consumption zones and a major suture between the northern and southern plates(3, 8).
The geological samples used in this study were collected from a wide range of potential source areas across East Asia, each with distinct tectonic settings and geological characteristics (Fig. S1). Processes such as mid-ocean ridge spreading, plate divergence, convergence, and lateral displacement have shaped the evolution of the Earth’s surface and controlled tectonic activity, exerting varying influences on the products of geological processes.
In this research, geological samples from 27 potential carnelian source areas across Asia were collected to build a geochemical database for provenance studies. This database provides a comparative framework for sourcing carnelian beads from the Sanxingdui sacrificial pits and related archaeological sites. Based on the tectonic subdivision of Asia, the sampled source areas include seven in the South Asian subcontinent, six in the North China craton, seven in the Yangtze-Cathaysia block, five in the Central Asian Orogenic Belt, and two in the Kunlun-Qinling-Dabie orogenic belt (Fig. S1).

Sanxingdui Site

Sanxingdui represents one of the most important Bronze Age sites in the upper reaches of Yangtze River and provides a crucial counterpoint to the well-documented sociopolitical developments of the Central Plains, China. Spanned ca. 1700–1000 BCE, the site flourished contemporaneously with the Erligang and Late Shang cultures but displays a distinctive material tradition, including monumental bronze masks, bronze figures, gold objects, ivory, and elaborately worked jades. These features indicate the emergence of a powerful regional polity in the upper Yangtze basin, whose political economy and ritual system diverged from the bronze-based state traditions of northern China(9, 10). Although Sanxingdui shares certain artistic and technological traits with

neighboring regions, the scale, composition, and ideological role of its ritual assemblages underscore its position as an independent cultural center embedded within wider interregional networks.

The eight sacrificial pits uncovered at Sanxingdui form one of the most extraordinary ritual deposits known in Bronze Age East Asia. These pits were intentionally excavated and rapidly filled with fragmented and burned objects, including bronzes, jades, gold items, elephant tusks, cowrie shells, and other high-value materials. Stratigraphic evidence and radiocarbon dates indicate at least two large-scale episodes of ritual deposition between ca. 1200 and 1000 BCE. The deliberate fragmentation, burning, and mixing of objects reflect highly formalized ritual practices, possibly associated with political transformation or renewal of cultic authority. Because the pits contain curated elite objects rather than ordinary domestic refuse, they offer a uniquely preserved cross-section of the materials that circulated within the highest levels of Sanxingdui society(11).

The materials deposited in the pits -- bronzes, gold, jade, ivory, and exotic stones -- represent the most valued categories of ritual paraphernalia within the Sanxingdui elite group. Carnelian beads occur only in these ritual deposits and not in general settlement contexts, underscoring their symbolic and social importance. By determining the geological origins of the carnelian from the pits, we gain direct insight into the long-distance acquisition networks mobilized by the Sanxingdui elite. Such data offer an independent line of evidence for interregional interaction, complementing previous archaeological, technological, and genetic research. The incorporation of non-local raw materials into Sanxingdui's ritual system provides a critical window onto the mechanisms of prestige accumulation, political authority, and cultural connectivity during a formative period of early state development in the region.

Carnelian beads presented in the manuscript
Sanxingdui sacrificial pits (n = 11)
The eight sacrificial pits at Sanxingdui date to approximately 3200-3000 yrBP, corresponding roughly to the late Shang to early Western Zhou periods(12).
Four carnelian samples from Sacrificial Pit K2 were found inside a bronze vessel *Lei*(9).
One carnelian bead was recovered from the basal burial deposits of Pit K5(10). Two carnelian beads from Pit K7 were unearthed beneath the ivory layer, scattered among bronze artifacts, jade objects, and small quantities of gold items(13). Four carnelian beads from Pit K8 were mainly distributed within the ash layer, deposited together with small bronze artifacts, gold objects, jade pieces, and fragments of large bronze vessels(14).

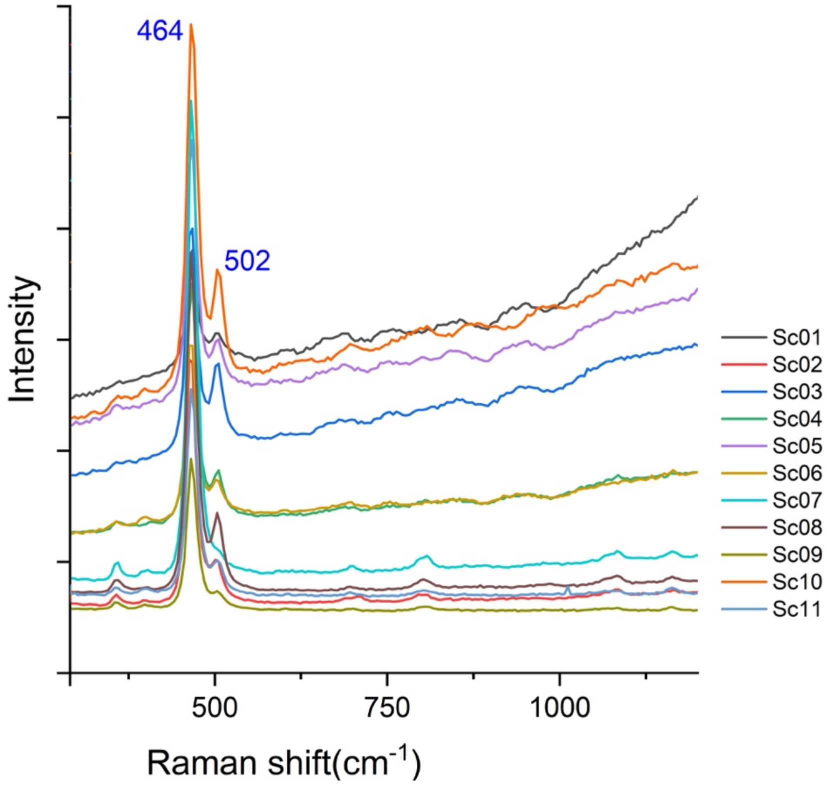

Fig. S2 Raman spectrum of the chalcedony beads unearthed from Sanxingdui. The peak at 464 cm^{-1} corresponds to the strongest peak of α-quartz, while the peak at 502 cm^{-1} corresponds to the characteristic peak of moganite.

Mogou site (n = 8)

The Mogou site, located in Lintan County, Gansu Province, is primarily associated with the Qijia culture, with a small number of Siwa culture burials(15, 16). At Mogou, approximately 887 Qijia and Siwa burials were excavated in 2008–2009, yet the eight carnelian beads reported in this study derive from only seven graves—fewer than 1% of the total. It dates to 1750–1100 BCE. Eight carnelian beads were recovered from seven burials. With the exception of burial M720 (Siwa culture), all belong to the Qijia culture. Two beads were found near the skulls of individuals in burials M393 and M720, respectively; two beads were found near the necks of individuals in burials M355 and M475; one bead was found in the southern side chamber of burial M390; two beads were recovered 3 cm south of individual R1 at the base of burial M455; and one bead was found near the ear of individual R3 in burial M459.

Xingong site (n = 3)

The Xingong site, Beijing, dated back to 1500-1300 BCE, is a large, double-ditch-ring settlement site of the Datuotou culture(17). Only three carnelian items were recovered from 27 Shang-period burials (two from tombs, M169 and M77, and one from a refuse pit K1), representing just 7.4% of the excavated graves; notably, the bead from M77 was found together with the only gold artifact (a gold earring) uncovered at the cemetery.

Zhaigou site (n = 2)

The Zhaigou Yutaliang cemetery in Qingjian County, Shaanxi Province, is a

small-scale Shang period site. At the cemetery, 34 Shang-period burials were excavated, dating to 1227-1000 BCE(18), and only one (M8) yielded carnelian beads—an incidence of 2.94%. Although the occupant of M8 was approximately four years old, the tomb was nearly adult-sized and was the only children's burial in the cemetery furnished with grave goods. These beads were strung together with cowrie shells and conch shells to form a necklace, found around the neck of the burial's occupant(19).

Data Preprocessing

Offline reduction of the analytical data—including selection of sample and blank signal intervals, correction for instrumental sensitivity drift, and calculation of elemental concentrations—was performed using the software ICPMSDataCal. First, optimal integration intervals for blank signals and sample test signals were selected. Based on the specific characteristics of each sample's signal, a stable interval with high signal intensity was chosen, and anomalous peaks within the interval were removed. For this study, an integration time of 28 seconds was generally used. Next, multi-external standard calibration was carried out using reference materials NIST610, NIST612, BHVO-2G, BCR-2G, and BIR-1G, with element Si selected as the internal normalization element. Finally, the quantitative compositional data of the samples were output.

Data cleaning Elemental concentrations below the detection limit were replaced with zero. For each sample, the average value of two measured points was taken as its compositional data. Outliers with SiO_2 contents below 98% were removed. Oxide-form elemental data (Na, Mg, Al, Si, P, K, Ca, Ti, Mn, Fe) were converted into ppm to unify the data format.

Data transformation Before statistical analysis, the elemental data in the integrated dataset were log-transformed.

Statistical analysis of source-area data

Canonical discriminant analysis (CDA) method was used for the statistical analysis of the source-data. CDA uses multivariate data to maximize separation among predefined groups(20). Cross validation is done for those cases to explore the accuracy of the classification in the analysis. CDA will give a predicted group memberships (PGM) based on the proximity of the ungrouped sample to the group's center (centroid) in the multi-dimensional space. The 1st PGM represents the group closest to the unknown sample while the 2nd PGM is the second nearest match group. In this study, geological samples from individual potential source were regarded as the predefined groups. The carnelian/agate beads from every site were treated as the ungrouped sample and assigned to PGMs.

The statistical process was completed in IBM SPSS Statistics 26 through multiple updates of group definitions and iterative statistical calculations for CDA. Wilks' Lambda stepwise method was used to select the most discriminative elements for group differentiation in each CDA procedure, as well as the same statistical parameters. The F value was chosen as the criteria for the stepwise method to determine the entry or removal of variables which contribute to maximizing the differences among groups in statistical analysis, with 3.84 as F entry value while 2.71 as F removal value (Default

value). An equal prior probability was assumed for all groups. Within-groups covariance matrix was used for the statistical procedure.

Initially, data from each of the 27 potential source areas were tentatively treated as separate groups for statistical analysis. Results showed that treating each source area as an independent group did not maximize differentiation between them. Fortunately, this attempt suggested that geological background should be used as a grouping criterion.

Based on large-scale geological background of the East Asia, the investigated sources were geologically grouped into several regional sources. Accordingly, all sources were classified into 8 regional sources at the first attempt to combine. The 8 regional sources groups were: "India" group with IMB, MJ, IPT, IRTP, IRTT, IUDR; "CAOB" group (Central Asian Orogenic Belt) with CNH, CTH, SCH, DKU, CALS, CXK; "YSO" group (Yanshan Orogenic) with CLB, CLN, CXH, CBP, CXZ; "GS" group (Gansu, China) with CDG, CGN; "NJ" group (Nanjing, China) with CNY; "Ytz" group (Yangtze Plate) with CBS, CMG, CZY, CBSh, CLS; "Cathy" (Cathy Plate) group with CPJ and "Bengal" group with BRM. CDA used 17 elements—Li, Be, B, Na, Mg, V, Mn, Fe, Cu, Ga, Sr, Y, Zr, Sn, Sb, Ba, U to derive corresponding discriminant functions for statistical analysis. The cross validation success rate for CDA was 86.3%. According to the result, India, CAOB and YSO groups were separated relatively. Bengal was closer to India than to Ytz, which shows the similarity between the sources of Bengal and India compared to the Yangtze Plate. The regional sources from South China (SC), Ytz, Cathay and NJ group were not well separated, which may indicate the similarity between them, or it may be caused by improper grouping conditions. GS group showed the same issue. In summary, South Asia (SA, Indian and Bengal), Central Asian Orogenic Belt, Yanshan Orogenic region sources were identified while other sources were still ambiguous at this step.

In the second step, source regions from South China were redefined and regrouped. The possibility of the separation between samples from Liangshan in Sichuan Province and Baoshan in Yunnan Province were discussed. Raw materials from Liangshan and Baoshan are considered separately, while the former is related to Permian Emeishan LIP (EM) and the later to Cenozoic Indo-Asian Collision (IEAC). At the same time, the India and Bengal region sources were combined for CDA. GS, NJ and Cathy groups were still considered individually. All sources were redefined into 8 source groups: "SA" group with BRM, IMB, MJ, IPT, IRTP, IRTT, IUDR; "IEAC" group with CBS, CBSh; "CAOB" group with CNH, CTH, SCH, DKU, CALS, CXK; "YSO" group with CLB, CLN, CXH, CBP, CXZ; "GS" group with CDG, CGN; "NJ" group with CNY;"EM" group with CMG, CZY, CLS and "Cathy" group with CPJ. CDA used 14 elements—Li, Be, Na, Mg, V, Mn, Fe, Cu, Ga, Sr, Y, Sb, Ba, U to derive corresponding discriminant functions for statistical analysis. The cross validation success rate for CDA was 81.3%. As can be seen from the statistical result, the sources located in southern China were not well separated as before by changing the grouping conditions, which guided to the combination of southern China in the next step. The separation of sources in southern China requires further exploration in the future. SC group was thus integrated and identified at this stage while GS still showed no sign of separation.

In the third step, sources in Gansu are further discussed due to the non-ideal aggregation and separation in the previous analysis. Two sources from Gansu, CDG & CGN, are considered separately. All sources were classified into 6 source groups as follows, "SA" group with BRM, IMB, MJ, IPT, IRTP, IRTT, IUDR; "SC" group with CBS, CMG, CZY, CBSh, CLS, CPJ, CNY; "CAOB" group with CNH, CTH, SCH, DKU, CALS, CXK; "YSO" group with CLB, CLN, CXH, CBP, CXZ; "CDG" group with CDG; "CGN" group with CGN. CDA used 19 elements—Li, Be, Na, Mg, Si, P, Ca, V, Mn, Cu, Ga, Sr, Y, Nb, Sb, Cs, Ba, Pb, U to derive corresponding discriminant functions for statistical analysis. The cross validation success rate for CDA was 89.0%. According to the results of CDA, CDG was grouped into Southern China, while CGN into Central Asian Orogenic Belt.

Through iterative refinement of the grouping conditions and repeated statistical tests, the final grouping was established as follows:

1. South Asia (SA): BRM, IMB, MJ, IPT, IRTP, IRTT, IUDR
2. Southern China (SC): CBS, CMG, CZY, CBSH, CLS, CPJ, CNY, CDG
3. Central Asian Orogenic Belt (CAOB): CNH, CTH, SCH, DKU, CALS, CXK, CGN
4. Yanshan Orogen (YSO): CLB, CLN, CXH, CBP, CXZ

Based on this final grouping, canonical discriminant analysis (CDA) achieved a classification success rate of 92.3% for the original grouped cases and 90.3% for cross-validation, which is considered satisfactory at the regional level. A two-dimensional visualization of the statistical results is shown in Figure X. At this stage, reliable differentiation between grouped source areas was achieved.

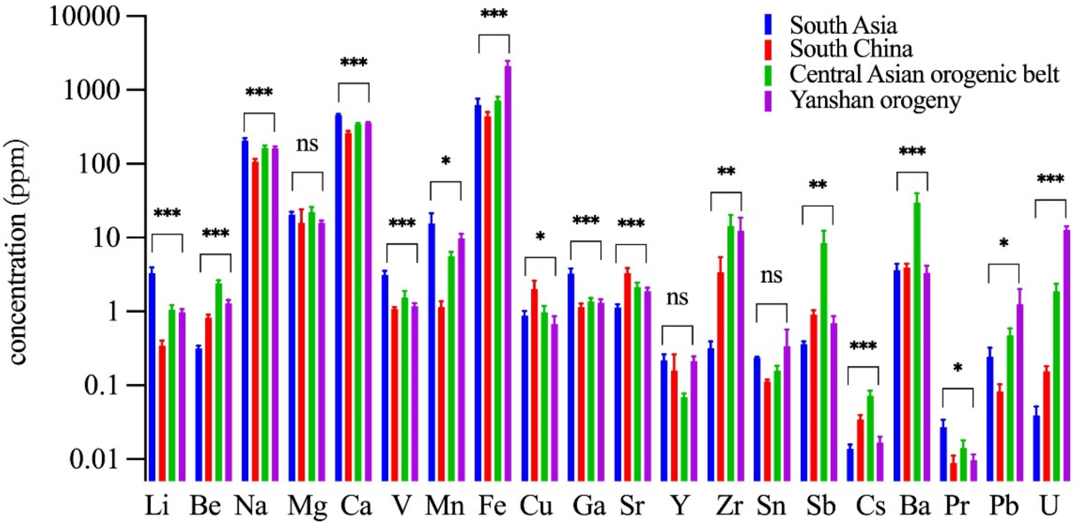

Fig. S3 Comparison of elemental compositions identified by CDA for the four potential sources

In this analysis, CDA used 20 elements—Li, Be, Na, Mg, Ca, V, Mn, Fe, Cu, Ga, Sr, Y, Zr, Sn, Sb, Cs, Ba, Pr, Pb, and U—and derived corresponding discriminant functions for the subsequent determination of archaeological samples.

Reference

1. R. Pabian, B. Jackson, P. Tandy, Agates: Treasures of the Earth (Firefly Books, 2006).
2. B. E. Luedtke, An Archaeologist's Guide to Chert and Flint (Cotsen Institute of Archaeology Press at UCLA, 1992).
3. L. Ma, X. Ding, L. Min, The Institute of Geology,Chinese Academy of Geological Sciences, Eds., Geological Atlas of China (Geological Publishing House, 2002).
4. S. Chatterjee, A. Goswami, C. R. Scotese, The longest voyage: Tectonic, magmatic, and paleoclimatic evolution of the Indian plate during its northward flight from Gondwana to Asia. Gondwana Research 23, 238–267 (2013).
5. J. Deng, et al., The Sequence of Magmatic-Tectonic Events and Orogenic Processes of the Yanshan Belt, North China. Acta Geologica Sinica (Eng) 78, 260–266 (2004).
6. Y. Xia, X. Xu, G. Zhao, L. Liu, Neoproterozoic active continental margin of the Cathaysia block: Evidence from geochronology, geochemistry, and Nd–Hf isotopes of igneous complexes. Precambrian Research 269, 195–216 (2015).
7. W. Xiao, et al., Accretionary processes and metallogenesis of the Central Asian Orogenic Belt: Advances and perspectives. Sci. China Earth Sci. 63, 329–361 (2020).
8. Q. Bian, et al., A Study of the Kunlun-Qilian-Qinling Suture System. Acta Geologica Sinica (Eng) 75, 364–374 (2001).
9. The Institute of Archaeology of Sichuan Province, Excavation of the Saccrificial Pits at Sanxingdui (Cultural Relics Press, 1999).
10. H. Ran, et al., Newly discovered sacrificial pits at the Sanxingdui site: Insights into Bronze Age ritual remains in Southwest China. Archaeological Research in Asia 42, 100621 (2025).
11. L. Von Falkenhausen, The external connections of Sanxingdui. Journal of East Asian Archaeology 5, 191–245 (2003).
12. Sichuan Province Institute of Cultural Relics and Archaeology, The Joint Laboratory of Archaeological Chronology of The Archaeological Research Center of the National Cultural Heritage Administration and School of Archaeology and Museology of Peking University, A C14 study on the sacrificial pit K4 of the Sanxingdui site Guanghan, Sichuan province. Sichuan Cultural Relics 117–120 (2021).
13. Sichuan Province Institute of Cultural Relics, Archaeology and School of Archaeology and Museology Sichuan University, Preliminary report on the excavation of sacrificial pit K7 at the Sanxingdui site in Guanghan, Sichuan. Sichuan Cultural Relics 25–45, 2, 121 (2025).
14. Sichuan Province Institute of Cultural Relics and Archaeology, School of Archaeology and Museology Peking University, Preliminary report on the excavation of sacrificial pit K8 at the Sanxingdui site in Guanghan, Sichuan. Sichuan Cultural Relics 21–42 (2024).
15. Gansu Provincial Institute of Cultural Relics and Archaeology, Center for Cultural Heritage and Archaeology Northwest University, A Qijia Cultural Cemetery, Mogou in Lintan County, Gansu Province. Archaeology 49, 10–17, 2, 100–103 (2009).

16. Gansu Provincial Institute of Cultural Relics and Archaeology, Center for the Protection and Archaeological Research of Silk Road Cultural Heritage Northwest University, Preliminary Report on the 2009 Excavation of Mogou cemetery of Siwa culture in Lintan County, Gansu Province. Cultral Relics 24–38, 1, 97–98 (2014).
17. J. Yang, W. Luo, H. Han, D. Chen, On-site analysis of the turquoise artifacts excavated from the Xingong site in Beijing. Journal of Archaeological Science: Reports 61, 104981 (2025).
18. Shaanxi Academy of Archaeology, School of Archaeology and Museology Peking University, Yulin Cultural Relics and Archaeological Survey Team, Yulin Institute for Cultural Relics Protection, Qingjian County Museum, Shang-Period Cemetery at the Zhaigou Site in Qingjian, Shaanxi. 考古 34–52, 2 (2024).
19. Shaanxi Academy of Archaeology, Yulin Cultural Relics and Archaeological Survey Team, Yulin Institute for Cultural Relics Protection, Qingjian County Museum, Preliminary Report on the Excavation of the Shang-Period Cemetery in the Yutaliang Locality at the Zhaigou Site in Qingjian, Shaanxi. Archaeology and Cultural Relics 70–79 (2024).
20. M. Baxter Jr., Exploratory multivariate analysis in archaeology (ISD LLC, 2015).
